# Understanding the mechanisms of drug-associated interstitial lung disease

**DOI:** 10.1038/sj.bjc.6602065

**Published:** 2004-08-31

**Authors:** T Higenbottam, K Kuwano, B Nemery, Y Fujita

**Affiliations:** 1 Global Clinical Science, AstraZeneca R&D, Charnwood, Bakewell Road, Loughborough, Leicester LE11 5RH, UK; 2 Research Institute for Diseases of the Chest, Graduate School of Medical Sciences, Kyushu University, Fukuoka, Japan; 3 Laboratory of Pneumology (Lung Toxicology), Katholieke Universiteit Leuven, Belgium; 4 Clinical Proteome Center, Tokyo Medical University, Tokyo, Japan

**Keywords:** gefitinib (‘Iressa’), EGFR-TKI, NSCLC, interstitial lung disease, pulmonary toxicity, proteomics

## Abstract

Drugs have been implicated in lung injury as a result of direct pharmacological action, persistence or metabolism in the tissue, or via the production of a reactive metabolite or metabolites. The result of this apparent drug-associated injury ranges from cellular dysfunction through to cell death (apoptosis) and alteration of repair mechanisms that are essential in replacing critical tissue elements and function. There is limited knowledge on how timing of drug administration or drug interactions may interfere with the repair mechanisms or modulate the expression of pulmonary toxicity. Chemotherapeutic drugs and novel agents, such as those targeting the epidermal growth factor receptor (EGFR), appear to affect both normal and neoplastic cells. However, unlike chemotherapy, where the actions are systemic and directly as a result of biotransformation or cell injury, it has been postulated that effects of EGFR-targeting agents are more likely to be focused on epithelia via a pharmacological effect. Furthermore, risk factors for the development of adverse pulmonary reactions, as well as biological markers indicating incipient toxicity, need to be prospectively identified. Proteomics, through the identification of ⩾1000 proteins or peptides in blood samples, will hopefully identify candidates for this role.

## INJURY AND REPAIR IN THE LUNGS

Pulmonary drug toxicity is increasingly being recognised as a cause of interstitial lung disease (ILD) and, as this lung disorder can be progressive and fatal, early recognition is important. However, drug-associated ILD can be difficult to diagnose, as we have seen in earlier sections of this supplement (see Camus *et al*, 2004 and Müller *et al*, 2004). There is a limited understanding of the mechanisms of drug-associated injury of the lung, compared with other tissues such as the liver, and no specific markers exist to differentiate drug-associated ILD from other pathological processes. Also, many drugs are used simultaneously or in close sequence and that makes assignment of toxicity to a specific agent difficult. In particular, ILD may be a comorbid condition of lung cancer, seen at the onset of disease and after many forms of chemotherapy. Numerous questions remain unanswered with this condition: in what manner do drugs or chemicals cause cell injury in the lung; why do they cause injury in the lung and not in other organs; why do they cause injury in only a minority of patients?

Alveolar and bronchial epithelial cells may be injured by inhalation of a drug or through the vascular system (Campbell and Senior, 1981; Kay, 1986; Shimabukuro *et al*, 2003). Acute injury seems to progress to chronic inflammation aided by T-lymphocytes and macrophages. Continued exposure to an antigen or failure of the lung's intrinsic anti-inflammatory mechanisms have been suspected as causes of persisting inflammation. Whatever the cause, this inflammation may in some instances lead to a fibrotic change that ultimately interferes with gas exchange. In some forms of ILD, the alveoli fill with lipoproteins, blood or eosinophils, in others there is an unexplained proliferation of smooth muscle mass, while still others result in amyloid deposits in the alveolus and the alveolar capillary membrane. Less extensive pathological changes are potentially reversible. The primary goal of treatment is to suppress the inflammatory response and reverse the deposition of granulation tissue.

In response to injury to the bronchial epithelium, there is an immediate requirement to initiate tissue repair and restore barrier function (Matthay, 1994). This involves migration of epithelial cells adjacent to the area of damage into the wound to form a temporary barrier consisting of poorly differentiated cells. However, as these cells are unlikely to perform the normal differentiated functions of epithelium, there follows a period of cell division and redifferentiation, leading to a complete restoration of normal epithelial barrier function.

### Epithelial apoptosis

Apoptosis, a feature of normal and injured alveolar epithelium, is a natural series of events in a cell that leads to its death. A substantial body of literature supports a role for apoptosis in the remodelling of lung tissue after acute lung injury for both the clearance of excess epithelium cells after repair (Bardales *et al*, 1996) and the normal removal of excess mesenchymal cells from resolving lesions (Polunovsky *et al*, 1993).

**Figure 1:**
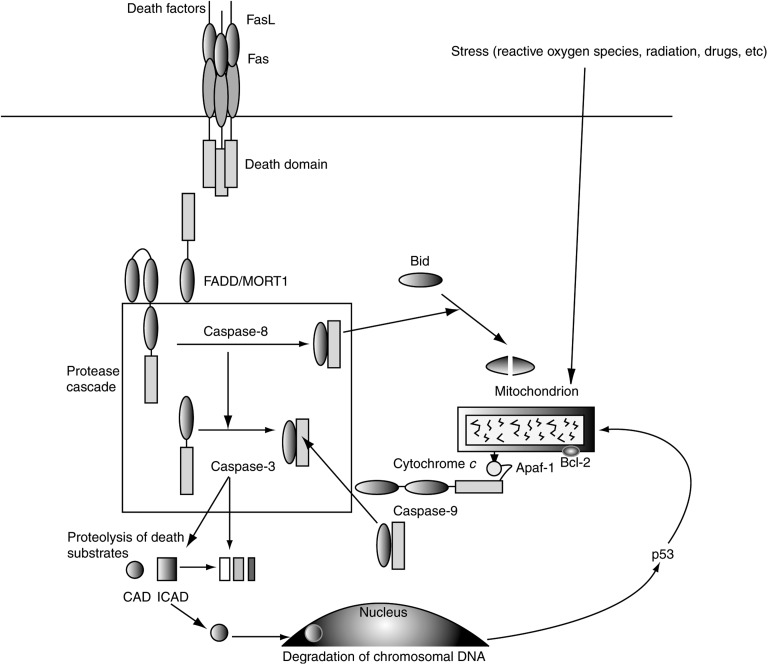
Apoptosis signalling pathways mediated by Fas or mitochondria (Kuwano *et al*, in press). Reprinted with permission from: Kuwano *et al* (in press). Epithelial cell apoptosis in lung injury and fibrosis (see Kuwano *et al*, in press). FasL=Fas ligand; FADD=Fas-associated death domain; MORT1=Mediator of receptor-induced toxicity 1; CAD=caspase-activated deoxyribonuclease; 1CAD=inhibitor of CAD; Apat-1=apoptotic protease activating factor-1.

Apoptosis pathways can be triggered by surface receptors, which interact with soluble proteins or membrane-bound proteins such as Fas ligand (FasL). Fas ligand is a cell surface molecule expressed predominantly in activated T-lymphocytes and natural killer cells (Suda *et al*, 1993) and is involved in the downregulation of immune reactions (Brunner *et al*, 1995; Ju *et al*, 1995). The activation of death receptors, such as Fas, result in the recruitment of adaptor proteins through interaction of a death domain (Figure 1

) (Kuwano *et al*, in press). Recruitment of Fas-associated death domain to Fas activates caspase-8.

Stimuli other than death receptor activation (e.g. anticancer drugs, radiation and reactive oxygen radicals) that can trigger apoptotic pathways initiate at the mitochondria. Cytochrome *c* is released into the cytosol from the mitochondria and binds to Apaf1 with adenosine triphosphate, resulting in the activation of caspase-9. The activation of caspase-8 or -9 leads to the activation of the caspase cascade. Fas ligand accumulates in soluble form at sites of tissue inflammation and has the potential to initiate apoptosis of leucocytes, epithelial cells and other parenchymal cells. It is possible that Fas, FasL, p53, p21 and other apoptosis-regulating proteins play an important role in the pathophysiology of lung injury and fibrosis. Animal studies have suggested that the Fas–FasL pathway plays an essential role in the development of pulmonary fibrosis and that inhibiting this pathway could have therapeutic value in lung injury and apoptosis (Hagimoto *et al*, 1997; Kuwano *et al*, 1999a). DNA damage or apoptosis has also been demonstrated in bronchial and alveolar epithelial cells in patients with idiopathic pulmonary fibrosis (Kuwano *et al*, 1996).

Although the mechanisms by which lung fibrosis develops are not fully understood, recent findings suggest that oxidative stress may play an important role in the pathogenesis of tissue fibrosis affecting apoptosis of both structural and inflammatory cells and altering the cytokine microenvironment balance. Indeed, a study that investigated oxidative stress and mitochondrial damage in lung epithelial cells from idiopathic interstitial pneumonias suggested that oxidative stress may participate in epithelial cell damage in idiopathic interstitial pneumonia (Kuwano *et al*, 2003).

Increased production of transforming growth factor beta (TGF-*β*) was demonstrated in epithelial cells of terminal airways and alveoli in idiopathic pulmonary fibrosis (Khalil *et al*, 1991). In addition to multiple effects on the process of fibrogenesis, TGF-*β*1 can induce apoptosis in various cells. The mechanism of TGF-*β*1-mediated apoptosis probably varies among cell types. TGF-*β*1 is a potent inducer of apoptosis through caspase-3 activation and the downregulation of p21. It is also an enhancer of Fas-mediated apoptosis of lung epithelial cells (Hagimoto *et al*, 2002). p21 regulates the activation of caspase-3 through the procaspase-3–p21 complex formation and protects human hepatoma cells from Fas-mediated apoptosis (Suzuki *et al*, 1998, 1999).

TGF-*β*, p53, p21 and Fas are predominantly localised in the epithelial cells of the terminal airways and alveoli. It has been speculated that these proteins and the Fas–FasL pathway, which may be linked to each other, regulate the proliferation and death of bronchial and alveolar epithelial cells (Kuwano *et al*, 1999b).

### Loss of stem cells from epithelial surfaces

Acute lung injury is usually characterised by significant damage to the alveolar epithelial barrier and efficient regeneration of an intact epithelium is crucial to restore normal function of this barrier. The type II pneumocyte is the stem cell of alveolar epithelium and is primarily responsible for re-epithelialisation and restoration of normal alveolar architecture. During normal cell turnover or lung damage, these cells divide and differentiate into the predominant alveolar type I pneumocyte (Uhal, 1997; Fehrenbach, 2001). Widespread loss of the integrity of the non-neoplastic type I and II pneumocytes appears to be the primary cause of diffuse alveolar damage, which is characteristic of acute interstitial pneumonia.

### Differential expression of the epidermal growth factor receptor (EGFR) in normal and injured tissues

Members of the epidermal growth factor (EGF) family (i.e. EGF, transforming growth factor alpha [TGF-*α*], heparin-binding EGF-like growth factor [HB-EGF], amphiregulin, betacellulin and epiregulin) are likely to be important regulators of epithelial repair by virtue of their ability to stimulate cell migration, proliferation, differentiation and survival. Indeed, EGF, HB-EGF and TGF-*α* are known to have a direct role in cutaneous wound healing (Lawrence and Diegelmann, 1994; McCarthy *et al*, 1996).

EGF belongs to a family of growth factors that exert their biological effects by binding to and activating the EGFR (c-erbB1). The EGF and EGFR play a pivotal role in maintenance and repair of epithelial tissues; however, little is known about c-erbB receptors and their ligands in human bronchial epithelium. Much of the work on the EGFR in the lung has been performed in the context of cancer, where elevated EGFR expression is a frequent observation. This has led to EGFR-targeted agents, such as the EGFR tyrosine kinase inhibitor (EGFR-TKI) gefitinib (‘Iressa’), being developed for the treatment of non-small-cell lung cancer (NSCLC). In normal adult lung, the distribution of EGF and EGFR has been demonstrated by immunohistochemistry, with expression observed in the basal cell layer of the bronchial epithelium (Aida *et al*, 1994; Yoneda, 1994; Polosa *et al*, 1999) and in occasional type II alveolar pneumocytes (Aida *et al*, 1994). As EGF signalling may represent an important mechanism that helps coordinate the process of recovery from lung injury (Polosa *et al*, 1999; Puddicombe *et al*, 2000), it is possible that EGFR inhibition will partly reduce the ability of pneumocytes to respond to lung injury. Indeed, there have been reports of pulmonary toxicity with the EGFR-TKIs gefitinib (Forsythe and Faulkner, 2003; Inoue *et al*, 2003) and erlotinib (Dragovich *et al*, 2003; Tan *et al*, 2003; Yamamoto *et al*, 2003), as discussed elsewhere in this supplement (see Camus *et al*, 2004).

## MECHANISMS OF DRUG-ASSOCIATED CELL INJURY

The pathogenesis of drug-associated cell injury usually involves the participation of toxic drug metabolites that either elicit an immune response or directly affect the biochemistry of the cell (Kaplowitz, 2002). Some molecules can also elicit an effect directly rather than through metabolites, although such direct effects appear much less common. Drug metabolites derived by biotransformation can be associated with a variety of biochemical events, such as oxidative stress, redox changes, covalent binding and lipid peroxidation, which are linked with cell dysfunction and ultimately cell death. Furthermore, many potent mutagens may react with DNA to form covalent adducts, which are associated with mutations in proto-oncogenes or tumour suppressor genes and initiate cancer.

### Biotransformation

The most common transformation of drugs is by oxidation, which can produce reactive oxygen species capable of causing cell injury. There are several defence systems against reactive oxygen species and these include enzymatic systems, which contain enzymes that can inactivate the active oxygen species, or nonenzymatic systems, which will scavenge these oxygen radicals.

If the defence systems against toxic oxygen species are overwhelmed, a condition known as oxidative stress arises. One outcome of oxidative stress is lipid peroxidation that may lead to cell dysfunction/death. Furthermore, inflammation and impaired repair may also occur. The classic example of drug-associated oxidative stress in the lung is paraquat. Paraquat, a quaternary nitrogen herbicide, is a highly toxic compound that causes intracellular oxidative stress with the production of reactive oxygen species (Lewis and Nemery, 1995). In addition, nicotinamide adenine dinucleotide phosphate is depleted and the antioxidant defence system is overwhelmed (Keeling and Smith, 1982).

The same or a similar mechanism is probably involved with nitrofurantoin and possibly bleomycin. This explains why hyperoxia may increase the damage because hyperoxia will increase the production of reactive oxygen species and also further deplete the defence systems. The presence of transition metals augments these reactions with reactive oxygen species as they are considered dependent on free iron.

### Cytochrome *P*450 (CYP) and glutathione *S*-transferase enzyme systems of the lungs

Cytochrome *P*450s comprise a superfamily of enzymes crucial for the oxidative, peroxidative and reductive metabolism of a diverse group of compounds, such as endobiotics, bile acids, fatty acids, prostaglandins, leukotrienes and xenobiotics, including most therapeutic drugs and environmental pollutants (Nelson *et al*, 1996; Bertz and Granneman, 1997). These enzymes are predominantly present in the liver but lower levels have been detected in the lung (Nemery *et al*, 2001). Humans have been estimated to have at least 53 different CYP genes and the CYP1A1 (in smokers), 2B6, 2E1, 2J2 and 3A5 proteins are expressed in human lung.

The cell-specific localisation of individual CYP enzymes in the lung is poorly understood. However, according to immunohistochemical analyses, expression is widespread, occurring in various structures in bronchial and bronchiolar epithelium, Clara cells, alveolar lining cells and alveolar macrophages (Hall *et al*, 1989). CYP1A1 is mainly expressed in the epithelium of the peripheral airways (bronchiolar, terminal bronchiolar and alveolar epithelium) and is only observed in the lungs of smokers (Anttila *et al*, 1991). The most important other CYP enzyme identified in the lungs is CYP3A, localised to bronchial, bronchiolar and alveolar epithelium as well as alveolar macrophages.

Acetaminophen (paracetamol) is a common drug and can be conjugated in the liver to sulphate or glucuronic acid. If concentrations are too high and the conjugations are overwhelmed (e.g. by an overdose) oxidation occurs by CYP or possibly by prostaglandin synthase. Subsequently, reactive metabolites can covalently bind to proteins, leading to oxidative stress, or engage in toxic oxygen species production. Common drugs such as acetaminophen can also be metabolised in the lungs and probably cause damage or be associated with decreased antioxidant defence (Dimova *et al*, 2000).

### Differential metabolism in the lung

Some drugs are biotransformed differently in the lung from the liver. Indeed, there is a range of experimental pneumotoxic agents that have been shown to undergo specific metabolism in the lungs and hence cause injury selectively in the lung. For example, administration of naphthalene to male Swiss mice resulted in severe bronchiolar epithelial necrosis at doses at which hepatic and renal necrosis were not observed (Warren *et al*, 1982). Butylated hydroxytoluene (BHT) also causes pulmonary toxicity. Lungs from female mice injected intraperitoneally with BHT were injured (Marino and Mitchell, 1972).

A toxic metabolite may be produced when the lung is responsible for metabolism compared to the liver or, alternatively, if there is failure of detoxification of the active metabolite in the lung compared to the liver; these differences are often highly species-specific.

### Individual susceptibility

Drug toxicity may simply be a question of dose, and a high dose will cause a pharmacological-type reaction. Thus, drug toxicity may arise from ‘therapeutic misadventures’, such as drug–drug, drug–diet or drug–environment interactions. Alternatively, drug toxicity may be unpredictable or idiosyncratic.

For instance, alterations in biotransformation can be acquired as a result of therapeutic misadventures. Antioxidants may become depleted, hence reducing the cell's ability to survive oxidative stress. Genetic deficiencies in drug metabolism enzymes may also lead to susceptibility to certain drugs that require detoxification. One example is the common genetic polymorphism in the drug metabolism enzyme *N*-acetyltransferase, which is responsible for metabolism of the antituberculosis therapy izoniazid. An anecdotal example of a genetic deficiency associated with lung toxicity was published as a case report (Ackerman *et al*, 1988). A child with partial monosomy 21, which leads to halving of superoxide dismutase levels in the blood and lungs, was exposed to nontoxic oxygen levels during an operation. The child suffered severe pulmonary oedema, possibly due to an increased lack of defence against toxic oxygen species.

## HOW MIGHT RADIATION, CHEMOTHERAPY AND EGFR INHIBITION BE ASSOCIATED WITH LUNG INJURY?

Standard treatments for lung cancer have been associated with ILD. Radiation can cause acute or chronic injury depending on dose, duration, pre-existing lung disease and concomitant steroid use (Abid *et al*, 2001; Aviram *et al*, 2001). There have also been reports of ILD thought to be associated with lung cancer chemotherapy, such as gemcitabine (Lilly, 2003), docetaxel (Kunitoh *et al*, 1996; Merad *et al*, 1997; Wang *et al*, 2001), paclitaxel (Furuse *et al*, 1997), irinotecan (Fukuoka *et al*, 1992) and vinorelbine (Furuse *et al*, 1994). Treatment of NSCLC with combination chemoradiotherapy has been associated with the development of ILD (Schweitzer *et al*, 1995; Reckzeh *et al*, 1996; Yamada *et al*, 1998; Robert *et al*, 1999; Aviram *et al*, 2001; Komaki *et al*, 2002) and, as described previously, EGFR-targeted agents may be associated with pulmonary toxicity.

**Figure 2:**
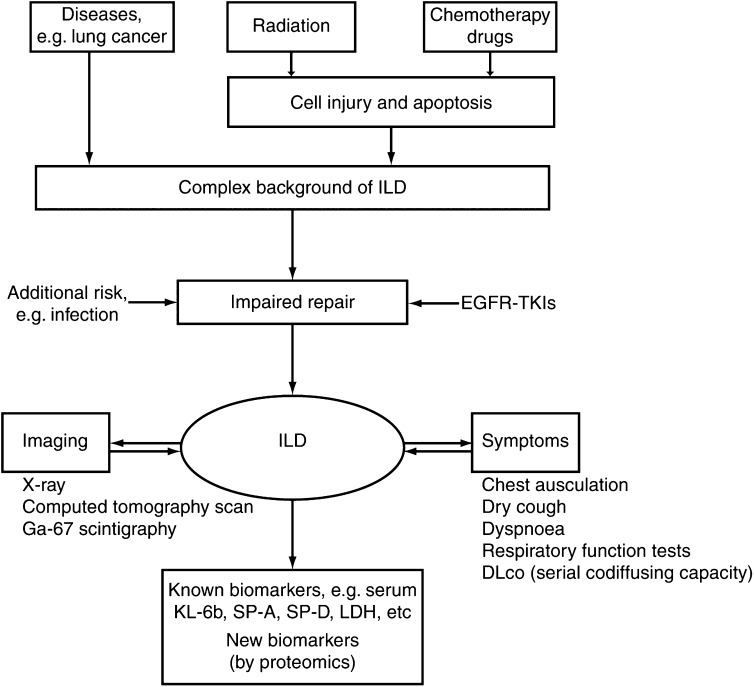
Development and diagnosis of ILD.

The development of ILD (Figure 2

) associated with anticancer agents is considered to be a multistep process, although one of the key initiating factors is likely to be the apoptosis of non-neoplastic type I and II pneumocytes. Mitochondrial-mediated apoptotic pathways, activated in lung tissues from patients with idiopathic interstitial pneumonia, may be involved in the pathophysiology of the disease (Kuwano *et al*, 2002). Radiotherapy and chemotherapy are unselective, damage DNA or alter repair, and will be likely to precondition the lung alveolar epithelium; however, as only a small fraction of patients develop ILD, it is likely that a further trigger factor, such as infection, is needed in preconditioned alveolar epithelium to cause ILD through impaired repair. While EGF signalling probably represents another potential mechanism that helps coordinate the process of recovery from lung injury by stimulating epithelial repopulation and restoration of barrier integrity (Aida *et al*, 1994), it is possible that EGFR inhibition, such as with gefitinib, will at least partly impair the ability of pneumocytes to respond to lung injury. Furthermore, it is very clear that any potential association of ILD with gefitinib is likely to be a pharmacological effect through EGFR on this repair process and not a result of biotransformation or chemical injury, as with radiotherapy and chemotherapy. However, to further understand the mechanisms involved in EGFR inhibition and ILD it would be interesting, through further research, to elucidate the key cell types involved (e.g. type II pneumocytes).

## PROTEOMICS AS A MEANS OF IDENTIFYING THE MECHANISM OF ILD AND POTENTIAL DIAGNOSTIC MARKERS FOR THE DISEASE

### Background of proteomics

The high incidence, severity and unpredictability of ‘chemotherapy lung’ has led many investigators to examine whether early detection of the disease is possible, using pulmonary function tests or imaging techniques (Wall *et al*, 1979; Castro *et al*, 1996; Cottin *et al*, 1996; Dawson *et al*, 2002). Some investigators believe it is prudent to discontinue chemotherapy once the diffusing capacity for carbon monoxide has dropped to ⩽50% of the pretherapy value (Castro *et al*, 1996). In contrast, others do not rely on this measurement since they believe that a decrease in the diffusing capacity does not represent toxicity in every patient (Bell *et al*, 1985; Magro *et al*, 1988); therefore, relying on this test may lead to unnecessary chemotherapy drug withdrawal. A rapid and consistent decrease in the diffusing capacity may indicate impending toxicity and requires careful examination of imaging data. When radiation therapy is planned after the completion of a chemotherapeutic schedule, it is advisable to wait for any chemotherapy-associated decrease in the diffusing capacity to stabilise or show a trend towards improvement before initiating radiation therapy. It is difficult to rely on imaging alone to detect pulmonary toxicity from chemotherapeutic agents, especially in patients with a background of changes resulting from lung cancer. Currently, there is no agreement as to how patients on chemotherapy should be followed using pulmonary function tests and imaging to reliably and cost-effectively detect therapy-associated pulmonary complications. Accordingly, attention is directed towards biological markers of early injury or disease.

Proteomics is a rapidly developing area of biomedical research that acts as a powerful tool for comprehensive and quantitative study of dynamic changes in proteins expressed from cells, tissues or organs under a variety of conditions. It also includes studies of protein–protein/protein–gene interactions, post-translational modifications such as phosphorylation or glycosylation, functions or cellular localisation. There are three components in this technology: (1) separation of mixtures of proteins/peptides (multidimensional chromatography); (2) high-sensitivity and high-throughput mass spectometry; and (3) database searching and annotation. Two-dimensional electrophoresis has been the most popular technology used for proteomic analysis, combining isoelectric protein migration in one dimension and size-determined separation in the second dimension. This procedure can resolve hundreds of components on a single gel. While it has limitations, such as lack of sensitivity for low abundant proteins, throughput or quantitativeness, there have been revolutionary improvements in the sensitivity of mass spectrometry, such as matrix-assisted laser desorption/ionisation time of flight, electrospray ionisation tandem mass spectrometry (ESI MS/MS) or surface-enhanced laser desorption–ionisation. Combination of microcapillary reverse-phase liquid chromatography with ESI MS/MS allows for an automated, high-sensitive and quantitative analysis of peptide mixtures.

The importance of proteomics reflects the major role of proteins in disease and the fact that proteins constitute a wide range of drug targets under active research. By studying protein interaction after drug treatment, proteomics may be used to determine the pathophysiological basis of disease and further investigate a drug's mechanism of action and toxicity. Moreover, proteomics can identify highly sensitive, specific protein markers and is a convenient and accurate method for identifying patients susceptible to diseases such as ILD. Furthermore, proteomics may detect patients presenting in early stages of disease and those most likely to respond to treatment.

### Use of proteomics in clinical interventions

Proteomics studies have been conducted on bronchiolar or nasal lavage fluid (Lindahl *et al*, 1999). However, as large amounts of protein present in sputum play important roles in normal lung physiology or the pathophysiology of lung disease, it is likely that proteomics will be increasingly used on sputum samples to evaluate the roles of different proteins in disease (Vignola *et al*, 2002). Furthermore, proteomics used for investigating the proteins involved in tissue repair and destruction may allow for better characterisation of the extent of airway remodelling in each patient (Vignola *et al*, 2002).

The principle of utilising proteomics as a method to obtain surrogate markers for ILD could use a limited number of patient enrolments and the creation of a panel of covarying proteins with disease phenotypes. Hundreds of precandidate proteins can be reduced to manageable numbers and statistical analysis and annotation can help elucidate the physiological roles of the selected proteins in terms of mode of actions of ILD. The selected protein panel (or protein chip) can then be validated through a blind test of large-scale patient populations.

### Relationship of proteomics to standard biomarkers of disease

Proteomics can be applied for the mechanistic study of drug actions by monitoring the phenotypic changes of cell lines and the corresponding protein expressions in a time-dependent manner. For example, if 500 serum samples of cancer patients are investigated, the top 30–50 up- or downregulated proteins of significance can be selected in correlation with cancer phenotypes, development stage, metastasis or ILD. Although the majority of these proteins will be known biomarkers of disease (e.g. SP-D, LDH, MCP-1), biomarkers involved in apoptosis and the cell cycle and so on, it is possible to discover novel proteins related to a drug response or drug resistance.

Hypothetically, almost all diseases are the result of imbalance in protein expression and by identifying specific profiles using molecular biology techniques we will have a better understanding of disease processes, therefore aiding in the development of differential diagnostic antibody chips for lung diseases.

## SUMMARY

It is well recognised that many agents, radiotherapy and a range of diseases, can cause ILD as a complication. Novel EGFR-targeted agents, such as gefitinib and erlotinib, may contribute an additional risk to the development of ILD, albeit via a pharmacological effect through the EGFR rather than directly as a result of biotransformation or chemical injury. Although a comprehensive knowledge of the mechanisms involved in the development of drug-associated ILD has not yet been achieved, the recent advances in genomics and proteomics may provide an opportunity to further develop the understanding of this complicated condition.
